# Characterization of Polysaccharide A Response Reveals Interferon Responsive Gene Signature and Immunomodulatory Marker Expression

**DOI:** 10.3389/fimmu.2020.556813

**Published:** 2020-10-26

**Authors:** Carlos A. Alvarez, Mark B. Jones, John Hambor, Brian A. Cobb

**Affiliations:** ^1^ Department of Pathology, School of Medicine, Case Western Reserve University, Cleveland, OH, United States; ^2^ Research Beyond Borders, Boehringer Ingelheim Pharmaceuticals, Ridgefield, CT, United States

**Keywords:** polysaccharides, T cells, interferon, co-regulatory receptors, microbiota

## Abstract

Polysaccharide A (PSA), a capsular carbohydrate from the commensal gut bacteria *Bacteroides fragilis*, has been shown to possess both potent T cell-dependent pro- and anti-inflammatory properties. PSA is able to induce abscess and adhesion formation in sepsis models, but can also inhibit asthma, inflammatory bowel disease (IBD) and experimental autoimmune encephalomyelitis (EAE) through MHCII-dependent activation of CD4^+^ T cells. Yet, despite decades of study, the ability of PSA to balance both these pro- and anti-inflammatory responses remains poorly understood. Here, we utilized an unbiased systems immunology approach consisting of RNAseq transcriptomics, high-throughput flow cytometry, and Luminex analysis to characterize the full impact of PSA-mediated stimulation of CD4^+^ T cells. We found that exposure to PSA resulted in the upregulation and secretion of IFNγ, TNFα, IL-6, and CXCL10, consistent with an interferon responsive gene (IRG) signature. Importantly, PSA stimulation also led to expression of immune checkpoint markers Lag3, Tim3, and, especially, PD1, which were also enriched and sustained in the gut associated lymphoid tissue of PSA-exposed mice. Taken together, PSA responding cells display an unusual mixture of pro-inflammatory cytokines and anti-inflammatory surface receptors, consistent with the ability to both cause and inhibit inflammatory disease.

## Introduction

Humans have evolved a complex relationship with colonizing bacteria in which the bacteria and their components play a key role in establishing tolerance and maintaining homeostasis. Indeed, dysbiosis of the gut microbiome is linked to autoimmunity (1, 2), hyper responsive disorders (3), and cancer development (4, 5), while use of commensal bacteria or their components can reduce disease burden (6–9).

*Bacteroides fragilis* is a gram negative and naturally occurring member of the normal human microbiota and has been robustly demonstrated to have both pro- (10) and anti-inflammatory (7, 11, 12) effects in rodents. This activity is mediated primarily by its capsular carbohydrate polysaccharide A (PSA) through its ability to elicit a strong T cell response (13) following processing *via* TLR2-stimulated nitric oxide production (14) and subsequent presentation by canonical class II MHC (MHCII) in a glycosylation-dependent fashion (15, 16). Interestingly, *B. fragilis* was originally identified and characterized as the most common anaerobic isolate from intra-abdominal abscesses (17), with the capsular complex being the key T cell stimulator required for this inflammatory and fibrotic response (13). However, it was later discovered that PSA could also prevent those same abscesses if the animal was expose to purified PSA prior to abscess induction (18). This initial discovery of an anti-inflammatory role has now been expanded to include the ability to protect against inflammatory models such as adhesion formation (19), asthma (11), IBD (20), and EAE (7).

Research focused upon the PSA-responsive T cell population has established that PSA exposure leads to the clonal expansion (21) of a subset of CD4^+^CD45Rb^low^CD62L^−^CD44^+^FoxP3^−^ T effector/memory (Rb^Lo^Tem) cells (12, 22). These cells suppress asthmatic inflammation in an IL-10–dependent fashion in cooperation with tissue-resident regulatory T cells (Tregs), whereby Tregs are selectively induced to release IL-10 when Rb^Lo^Tem cells are present (12, 22); however, the PSA-stimulated Rb^Lo^Tem cells are also strong producers of IFNγ, the canonical pro-inflammatory cytokine of T helper type 1 cells (Th1) (23). Given the acknowledged importance of the microbiota in the maintenance of homeostasis, and the potent and complex T cell stimulatory properties of PSA, we sought to generate a more complete characterization of the T cells activated and expanded by PSA through a combination of deep RNA sequencing transcriptomics, high throughput flow cytometric quantitation of surface proteins, and multianalyte Luminex analyses of secreted cytokines and chemokines.

We found that PSA induces the upregulation and expression of numerous immunological genes and molecules associated with an interferon responsive gene (IRG) signature, as well as transcription factors such as T-bet, signal transducer and activator of transcription (STAT) 1, and STAT4 which are associated with a Th1 phenotype (23, 24). Interestingly, T cell surface marker examination revealed the upregulation of immune-regulatory markers such as Tim3, Lag3, and especially PD1 in response to PSA, collectively pointing toward a regulatory phenotype. Together with *in vivo* validation of these changes within the gut-associated lymphoid tissue (GALT) of orally PSA-exposed mice, these results show the PSA response is primarily driven by interferon but is phenotypically both anti- and pro-inflammatory, suggesting that the overall immunologic outcome of PSA exposure is highly context dependent. These data help to better understand the true nature of our relationship with the glycome of commensal microbes like *B. fragilis* which are known to provide strong systemic immunologic benefit under homeostatic circumstances (7, 9, 11, 18, 22, 25), while causing inflammation if they breach the normal gut compartment (6, 10, 17).

## Materials and Methods

### Mice

C57BL/6J (Stock #000664), FoxP3 RFP (C57BL/6-FoxP3 tm1Flv/J, Stock #008374 mice, all on the C57BL/6 background were purchased from the Jackson Laboratory (Bar Harbor, ME). Mice were housed in a 12-h light/dark cycle specific pathogen free facility and fed standard chow (Purina 5010) ad libitum. Enrichment and privacy were provided in mating cages by “breeding huts” (Bio-Serv S3352-400). Mouse studies and all animal housing at Case Western Reserve University were approved by and performed according to the guidelines established by the Institutional Animal Care and Use Committee of CWRU.

### PSA Purification

PSA was isolated from 20 L of log-phase *B. fragilis* culture, using the NCTC9343 *B. fragilis*-derived Δ44 strain expressing only PSA (26) exactly as previously described (27). Purity was determined by SDS-PAGE, BCA assay for protein, and absorbance scans for protein and nucleic acid.

### Primary Splenic, Peyer’s Patch, and Mesenteric Lymph Node T Cells

#### In Vitro Experiments

Primary splenocytes were isolated from freshly harvested spleens, PPs or MLNs and reduced to a single cell suspension by passing them through a sterile 100µM nylon mesh cell strainer (Fisher Scientific, Hampton, NH). For splenic T cell sorting, single cell suspensions were labeled with anti-mouse CD4 magnetic microbeads (Miltenyi Biotec, San Diego, CA), and separated with an autoMACS Pro Separator (Miltenyi Biotec, San Diego, CA), per manufacturer’s instructions. Antigen presenting cells (APCs) among the CD4^−^ population were labeled with anti-mouse MHCII magnetic microbeads (Miltenyi Biotec, San Diego, CA) and separated using the autoMACS Pro Separator as before.

#### In Vivo PSA Exposure

100 µg of purified PSA dissolved in PBS was administered to mice with a blunt, rounded needle every 72 h for a total of five times.

### Cell Culture

#### PSA-Mediated Activation

100,000 CD4^+^ T cells were cultured in a 1:1 ratio with MHCII^+^ APCs in Advanced RPMI (Gibco/Fisher Scientific, Waltham, MA) supplemented with 5% Australian-produced heat-inactivated fetal bovine serum, 55 µM β-mercaptoethanol, 100 U/ml and 100 µg/ml Penicillin/Streptomycin, and 0.2 mM L-glutamine (Gibco/Fisher Scientific, Waltham, MA) at 5% CO_2_, 37°C. PSA was added at 50 μg/ml on day 0 and the cultures were allowed to incubate for the indicated amount of time.

#### IFNγ Stimulation

After flow sorting, cells were cultured in 96-well plates (Corning, Corning, NY) at 50,000 cells per type per well in Advanced RPMI as before. Recombinant mouse IFNγ was added at 10 ng/ml for 3 days prior to analysis of marker expression by flow cytometry.

### Flow Cytometry and Cell Sorting

For splenic, PP or MLN T cell flow cytometry, positively selected CD4^+^ cells were stained with combinations of antibodies (0.5 µg/ml per) to Tim3-APC (BioLegend, San Diego, CA) or Lag3-APC (BD Bioscience), PD1-APC (BioLegend, San Diego, CA), CTLA4-APC (BioLegend, San Diego, CA), Ly6A/E-APC (BD Biosciences, San Jose, CA), and GITR-APC (BD Biosciences, San Jose, CA). Cells were washed twice in MACS buffer (Miltenyi Biotec, San Diego, CA) before analysis using Attune NxT (ThermoFisher, Waltham, MA) with the support of the Cytometry & Imaging Microscopy Core Facility of the Case Comprehensive Cancer Center. Analysis of all FACS data was performed using FlowJo (Tree Star, Inc., Ashland, OR).

### ELISA, Blocking, Supplementation, and Luminex

Cytokine levels were analyzed by standard sandwich ELISA performed by manufacturer’s instructions (BioLegend, San Diego, CA), modified to utilize europium-conjugated streptavidin (Perkin-Elmer) detected with a Victor V3 plate reader (Perkin Elmer, San Jose, CA). Blocking experiments utilized antibodies to IFN-γ (10 µg/ml, BioLegend, San Diego, CA). For IFN-γ blocking experiments, indicated cell types were incubated with 10 µg/ml αIFN-γ at 4°C for 15 min, washed twice with PBS, then combined into co-culture. Supplementation assays were performed with recombinant mouse IFN-γ (BioLegend, San Diego, CA). For Luminex assays, media from indicated cultured populations were snap frozen in liquid nitrogen and sent to Eve Technologies (Calgary, Ontario, Canada) for mouse 32-plex and TGFβ 3-plex analysis.

### RNAseq and Analysis

For RNA sequencing, cells were harvested, re-purified from co-cultures using CD4 magnetic beads as before to a minimum of 95% purity with two passes through the autoMACS Pro system, and washed twice in PBS. Pelleted cells were snap-frozen in liquid nitrogen and shipped to LC Sciences, LLC. for extraction, RNA purification and quality check, library creation and high-throughput sequencing (Illumina). Differential expression analysis and GO was done using EdgeR v3.12.1 by LC Sciences, LLC. Genes showing significant differences (FDR >0.05 and log2CPM >0) were selected for enrichment analysis using GAGE v2.20.1 by LC Sciences, LLC. We acknowledge our use of the gene set enrichment analysis, GSEA software, and Molecular Signature Database (MSigDB) (28) (http://www.broad.mit.edu/gsea/). All RNAseq data has been deposited in the NCBI Gene Expression Omnibus (GEO) under the accession numbers GSE156042 and GSM4721206-GSM4721211.

### Data Analyses

All data are represented by mean ± SEM. Data and statistical measurements were generated with GraphPad Prism (v5.0). For comparisons between two groups, Student’s *t*-test was used; comparisons between multiple groups utilized analysis of variance.

## Results

### PSA-Activated T Cells Show Transcriptomic Changes Consistent With Clonal Expansion

We chose to examine the transcriptomic profile of PSA exposed murine CD4^+^ T cell splenocytes co-cultured with antigen presenting cells for 7 days *in vitro* (n = 3 per group). Using the least stringent analyses allowing for any significant (p < 0.05) non-zero (>0 fold log2CPM) change incorporating a false discovery rate (FDR) of <0.05, we identified over 16,000 differentially expressed genes (DEGs) relative to resting and unstimulated controls. Focusing upon the top 500 DEGs reveals clear groups of genes that are either up or down-regulated in response to PSA (**Figures S1A** and **Table S1**). Using a multi-dimensional scaling (MDS) plot to provide a global view of gene expression, we found a remarkably high degree of similarity between PSA-exposed replicates (**Figure S1B**), reflecting the clonal expansion and transcriptomic programming of T cells responding to PSA. In contrast, resting and unstimulated CD4^+^ T cells showed a much higher degree of heterogeneity, as expected (**Figure S1B**). We also performed gene ontology (GO) analysis, where we found that the most enriched terms were mitotic cell cycle process (GO:1903047, Biological Process; **Figure S1C**), pyrophosphatase activity (GO:0016462, Molecular Function; **Figure S1D**), and mitochondrial part (GO:0044429, Cellular Component; **Figure S1E**), collectively pointing to a metabolically active proliferative state.

**Figure 1:**
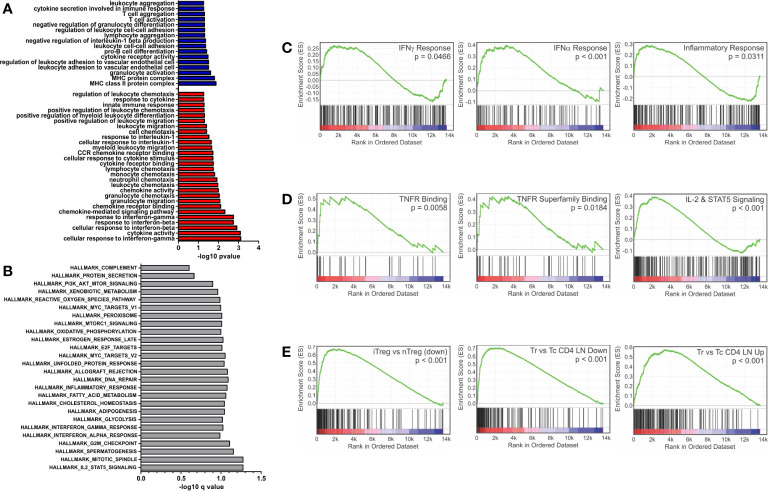
Gene set enrichment analysis of inflammatory and interferon related genes. Gene ontology and gene set enrichment analysis were conducted on differentially expressed genes (FDR > 0.05 and log2CPM > 0). **(A)** Gene ontology analysis of differentially expressed genes increased (red) and decreased (blue) with PSA exposure in CD4^+^ T cells. GO terms with immunological relevance were extracted (44 terms). Gene ontology terms significantly associated (P ≤ 0.05) were plotted as −log10 version. **(B)** Hallmark gene set enrichment analysis of PSA stimulated cells. Gene sets significantly associated (P ≤ 0.05) were plotted as −log10 of q value. GSEA analysis showing enrichment in **(C)** IFN-γ, IFN-α and inflammatory response gene sets, **(D)** TNF superfamily members and receptors, **(E)** and gene sets associated with Treg functionality.

Closer examination of GO terms revealed an enrichment of immunological functions such as the cellular response to IFNγ (GO:0071346), response to IFNβ (GO:0035458), cytokine activity (GO:0005125) and chemokine receptor binding (GO:0042379) (**Figure 1A**). Gene set enrichment analysis (GSEA) using the Hallmark gene sets (28, 29) and an FDR of <0.25 showed 27 enriched sets upon activation with PSA compared to control cells (**Figure 1B**). In parallel with the GO data, among the most highly enriched were interferon-α, interferon-γ and inflammatory response gene sets (**Figure 1C**). When compared to the immunological signature c7 gene set from the Broad Institute (28, 29), there was strong enrichment in sets associated with tumor necrosis factor receptor (TNFR) and TNFR super family binding as well as IL-2 and STAT5 signaling pathways (**Figure 1D**). Moreover, the DEG profile favors iTreg (**Figure 1E**, left) over both nTreg and T conventional (Tc) cell profiles (**Figure 1E**).

### PSA-Responding T Cells Show an Interferon Responsive Gene Signature

**Figure 2:**
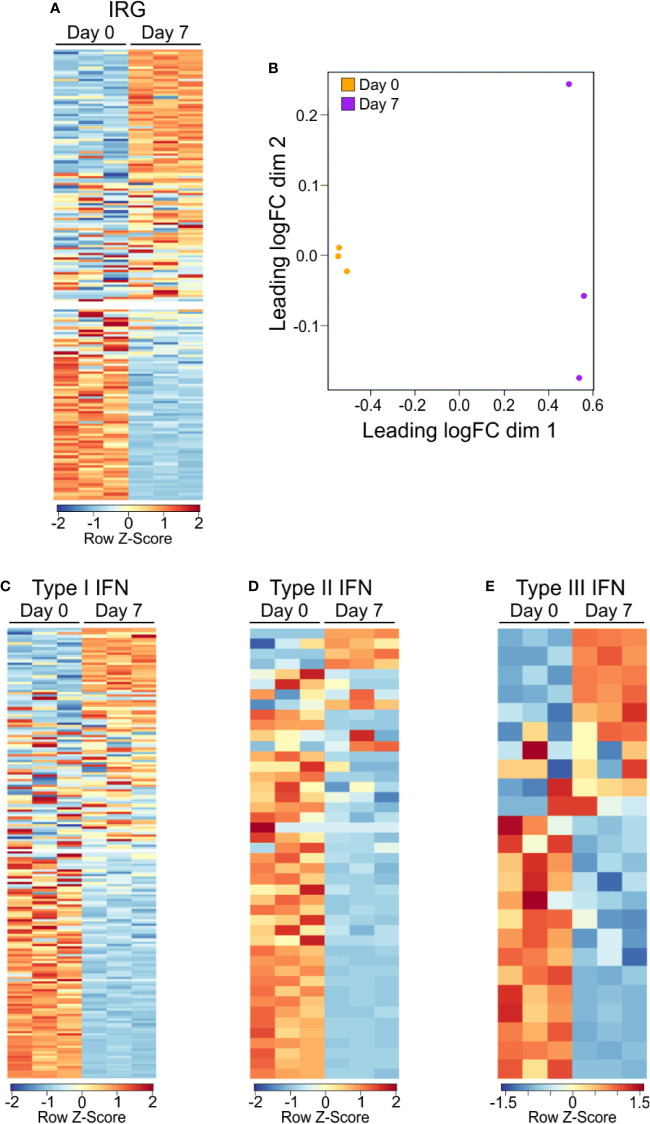
Gene expression IRG signature of PSA exposed cells. **(A)** Heat map of interferon responsive genes from PSA exposed (D7) or unexposed (D0) CD4^+^ T cells. Values are row z-score. **(B)** Multidimensional scatterplot of IRG data comparison of PSA exposed and non-exposed cells (D7 in orange, D0 in purple). **(C)** Breakdown of IRG list into Type I, Type II, or Type III IFN categories.

GO and GSEA analysis showed a consistent enrichment in immunologic and interferon signaling, whether as a direct or indirect result of exposure to interferon molecules (23, 30, 31). IRGs can be grouped depending on the type of interferon stimulation that induces their expression, including type 1 interferons (i.e., IFNα and IFNβ) which can be produced by almost any cell upon viral infection (32), type 2 interferon (IFNγ), which is secreted by a number of leukocytes to combat infectious agents or cancer (23), and type 3 interferon (i.e., IFNλ), which is secreted by both leukocytes and epithelial cells (33). In order to better categorize the PSA T cell response in terms of the apparent interferon response, we assembled of list of IRGs, including relevant cytokines, signaling molecules and markers associated with each type of interferon (**Table S2**). We found 128 of the 215 IRGs were differentially expressed between PSA-activated T cells and control T cells (**Figures 2A, B** and **Table S2**). Dividing the list into its associated IFN types further revealed clusters of upregulated and downregulated genes, although only IFNγ gene expression by RNA was observed (**Figures 2C–E**). This commonality in IRGs is likely due to the sharing of signaling machinery, such as Jak1/2, STAT1, and other IFNγ induced molecules, between the interferon types (31).

### PSA-Responding T Cells Show Th1-Skewed Signaling Molecule and Transcription Factor Expression

**Figure 3:**
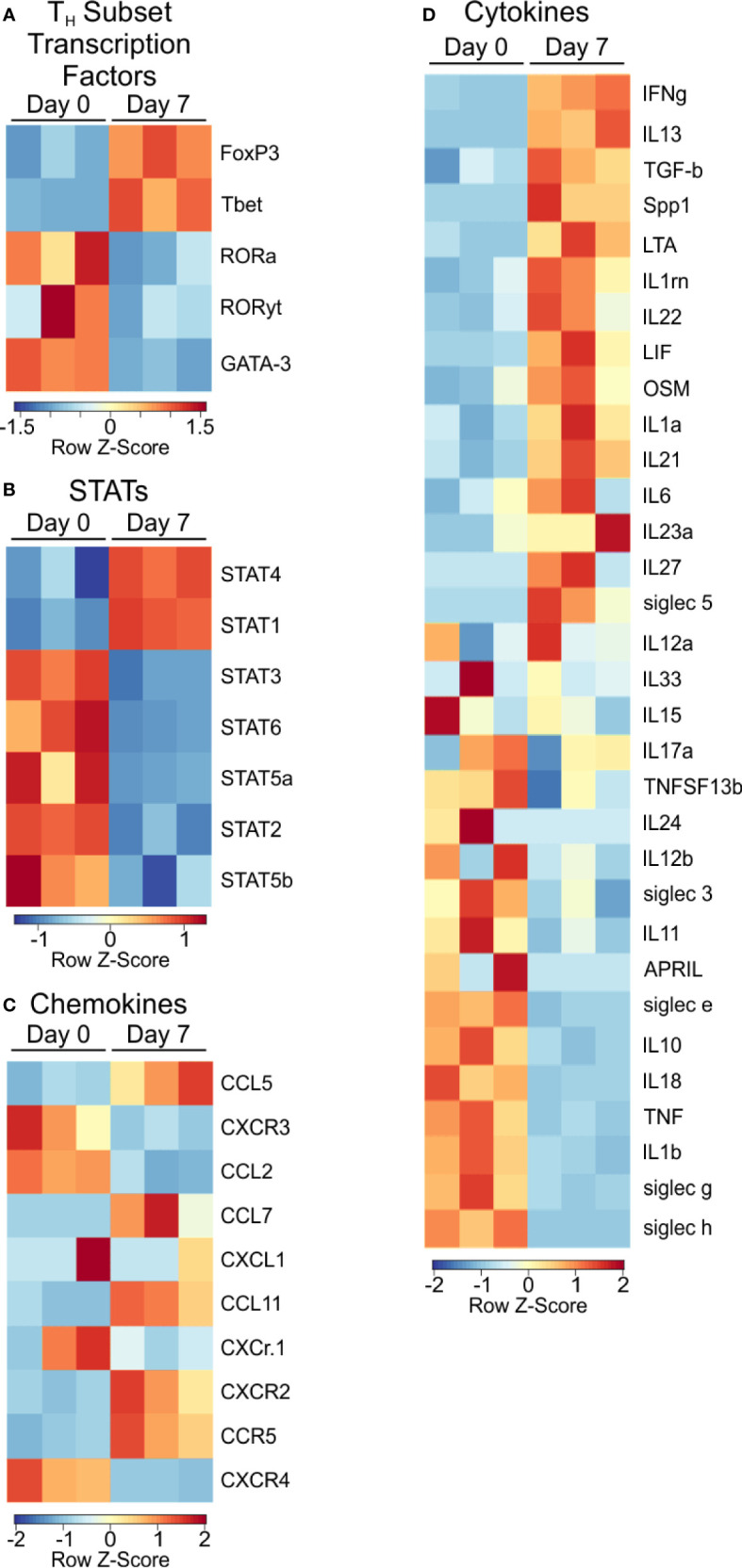
Transcription factor and signaling molecule profile of PSA exposed cells. Heat map of **(A)** T subtype associated transcription factors, **(B)** STAT transcription factors, **(C)** Chemokines, and **(D)** Cytokines of PSA exposed T cells compared to control.

Next, we examined signaling molecules such as transcription factors, chemokines and cytokines to identify the nature of PSA-mediated T cell skewing. Focusing on T cell lineage-associated transcription factors, we found T-bet and FoxP3, commonly associated with Th1 and Treg populations respectively, were transcriptionally upregulated (**Figure 3A**), yet FoxP3 protein was unchanged (**Figure S2**). Conversely, RORα, RORλt, and GATA-3 were not enriched in PSA-responding cells.

Examination of STAT molecules showed a selective upregulation of STAT1 and STAT4, both of which are closely associated with IFNγ signaling (**Figure 3B**) (24, 31). Similarly, the transcriptional expression of IFN inducible chemokines and receptors such as CXCR3, CCL5, and CCL2 (**Figure 3C**), combined with the upregulation of LIF, IL-1, and IL-6 (**Figure 3D**) are all consistent with the IRG signature of PSA.

### Protein Levels in PSA-Responding T Cells Are Consistent With an IRG Signature

Thus far, our analyses have relied solely upon transcript levels measured by RNAseq; however, it is well known that the correlation between gene transcription and protein concentration can be as little as 40%, depending on the system (34). This is driven by differences in mRNA transcription rate and accessibility, translational control, intracellular trafficking and metabolism (34). As a result, we first employed multianalyte Luminex analyses on secreted proteins from PSA-activated T cells and compared the result to the RNAseq data.

**Figure 4:**
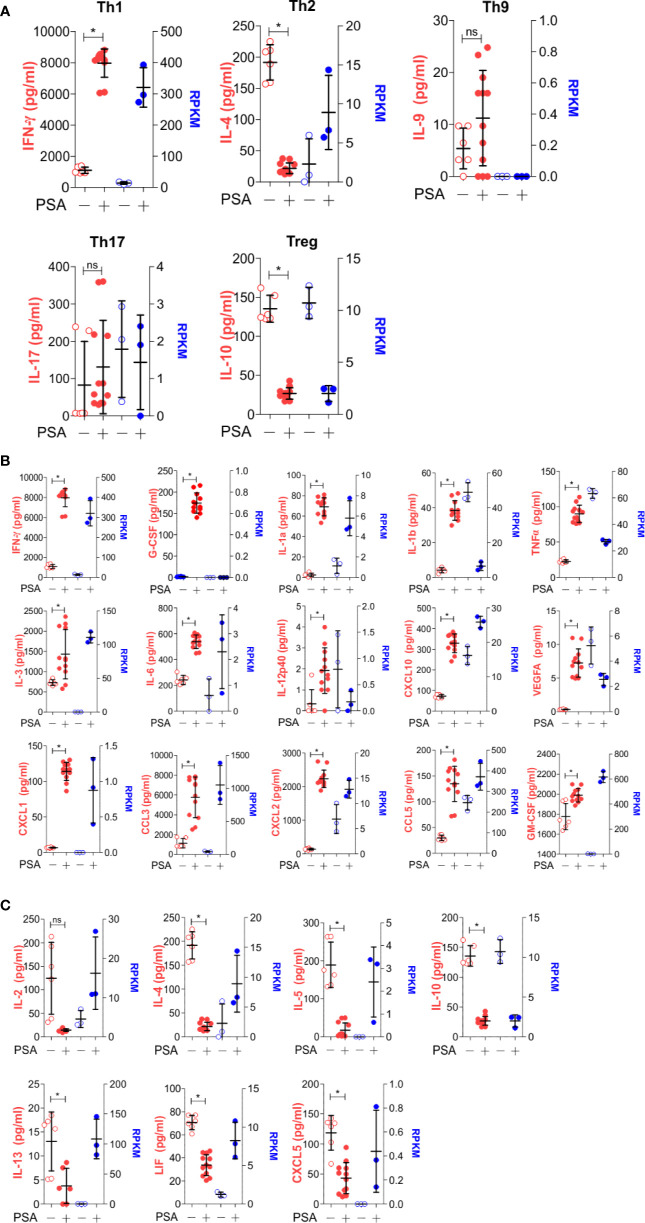
Secreted molecule profile of PSA exposed cells. Luminex analysis (Mouse Cytokine/Chemokine Array 32-plex) was used to determine secreted molecule profile of PSA exposed APC and T cell co-cultures after 7 days. Data shown reflects secreted proteins (left, red) and matching RNA-seq data (right, blue). **(A)** T subtyped associated cytokines. **(B)** Molecules that significantly increased (P ≤ 0.05) with PSA exposure. **(C)** Molecules that significantly decreased (P ≤ 0.05) with PSA exposure.

As before, T cell and APC co-cultures were setup with or without PSA for 7 days. Culture media from these co-cultures were used to quantify 32 cytokines, chemokines and growth factors. A comparison of canonical cytokines associated with specific T helper lineages (**Figure 4A**) showed robust IFNγ release that was mirrored in the level of IFNγ transcript from RNAseq, further supporting a Th1-skewed phenotype. Interestingly, IL-4 protein (Th2-associated) was reduced with PSA exposure at day 7, although transcript levels were increased, possibly suggesting that IL-4 is being released, but is being taken up by neighboring Treg cells (22), although no change in FoxP3^+^ cells or FoxP3 protein was apparent with or without IL-4 neutralization (**Figure S2**). For IL-9 (Th9) and IL-17 (Th17), both genes were not induced by PSA, while IL-10 (Treg) was reduced. Beyond these classical cytokines, we also found a number of other cytokines increased at the protein level, including IL-1, IL-6, IL-12, and CXCL10, although the mRNA transcript levels did not always reflect this increase (**Figure 4B**). Likewise, several cytokines were decreased in response to PSA at the protein level, including IL-2, IL-5, and IL-13 (**Figure 4C**), while still others did not change (**Figure S3**). As with IL-4, several molecules (i.e., IL-2, IL-5, IL-13, LIF, and CXCL5) were all increased at the mRNA level despite reductions in protein concentration.

**Figure 5:**
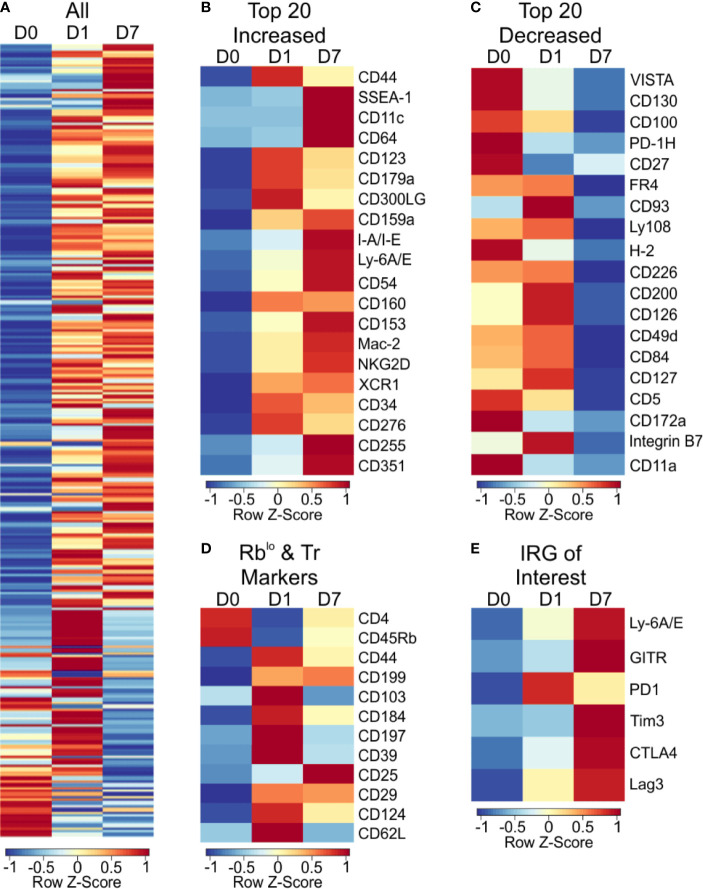
Cell surface marker expression of PSA exposed CD4+ T cells identifies immunomodulatory markers. High throughput flow cytometry was used to obtain the cell surface marker expression of 255 markers using LegendScreen Mouse PE kit. APC and T cells were co-cultured with PSA and collected at D0, D1, and D7. Data shown is geometric MFI from CD4^+^ T cells. Data is row z-scores. **(A)** Summary heat map of all 255 markers on D0, D1 and D7. **(B)** Top 20 markers most increased and **(C)** decreased on D7 compared to D0. **(D)** Markers previously used to identify PSA responsive T cells in human and murine experiments. **(E)** Activation and immunomodulatory markers upregulated with PSA exposure.

For broad coverage of cell surface molecules, we employed the LegendScreen platform (BioLegend) to quantify 255 target proteins by flow cytometry after 1 or 7 days of PSA-mediated activation. The global profile of all markers on CD4^+^ T cells at day 0, 1, and 7 calculated from the geometric mean fluorescence (GeoMFI) shows a distinctive pattern of expression at both days 1 and 7 (**Figure 5A** and **Table S3**). Examination of the top increased (**Figure 5B**) and decreased (**Figure 5C**) proteins revealed an increase in proliferation and activation markers such as CD44, Ly6A/E, and CD255 (35–37), and decrease in other activation markers such as CD100 and CD27 (38, 39). Interestingly, in addition to previously reported markers of PSA-expanded T cells (**Figure 5D**), the data revealed six regulatory receptors (co-regulatory receptors) not previously associated with the PSA response, including Tim3, Lag3, and PD-1 (**Figure 5E**).

### PSA-Associated Co-Regulatory Receptors Are IRGs

**Figure 6:**
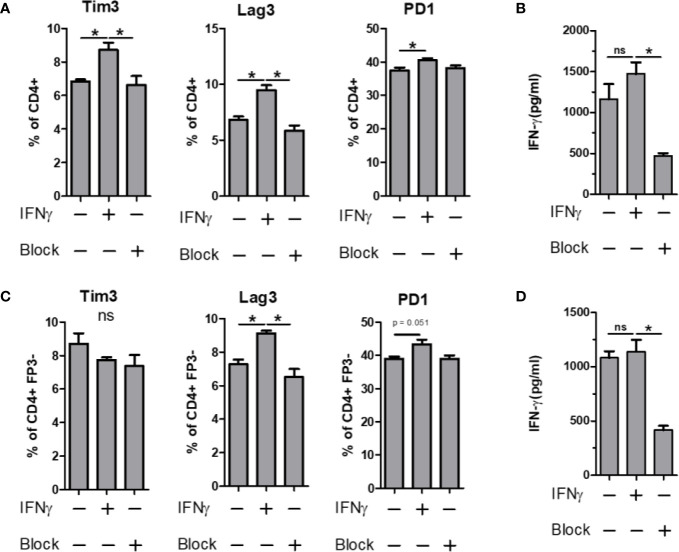
IFN-γ influence on immunomodulatory marker expression. Bulk or FP3^−^ CD4^+^ T cell were cultured *in vitro* for 3 days with α CD3 stimulation. Cells were supplemented with 10 ng/ml of recombinant mouse IFN-γ or with 10 µg/ml of α-IFN-γ blocking antibody. Marker expression assays with flow cytometry and culture supernatants were used for ELISA. Tim3, Lag3 and PD1 expression of **(A)** Bulk or **(C)** FP3^−^ CD4^+^ T cells with or without IFN or antibody blockade. ELISA data of culture supernatants from **(B)** Bulk or **(D)** FP3^−^ CD4^+^ T cell cultures. (significance of P ≤ 0.05 = *).

The expression of PD-1 is strongly associated with suppression of the immune response, a phenomenon exploited by several cancers (40, 41). Likewise, Tim3 and Lag3 have been shown to have important roles in immune suppression, usually in supportive roles of PD-1, leading to a synergistic upregulation of all three molecules (42, 43). Given the strong IRG signature of the PSA T cell response, we sought to determine whether IFNγ would induce the expression of these co-regulatory receptors in PSA-naïve T cells. CD4^+^ T cells, or flow sorted CD4^+^ FoxP3^−^ T cells were cultured in the presence of anti-CD3ϵ stimulation and supplemented with either recombinant IFNγ or anti-IFNγ neutralizing antibody for 3 days. We found that Tim3, Lag3, and PD-1 were all increased in response to IFNγ, and this increase was ablated in the presence of IFNγ-neutralizing antibody (**Figures 6A, B**). Moreover, this was true for Lag3 and PD-1 in both bulk CD4^+^ splenocytes (**Figures 6A, B**) and cultures lacking Tregs (**Figures 6C, D**). Tim3 increases were lost in T cell populations lacking Tregs. This suggests that changes in Lag3 and PD-1 are primarily in Tc cells, while Tim3 is primarily in Tregs. Reduced IFNγ in these experiments demonstrated the efficacy of the neutralizing antibody (**Figures 6B, D**).

### Co-Regulatory Receptors Are Increased Within the GALT of PSA-Exposed Mice

**Figure 7:**
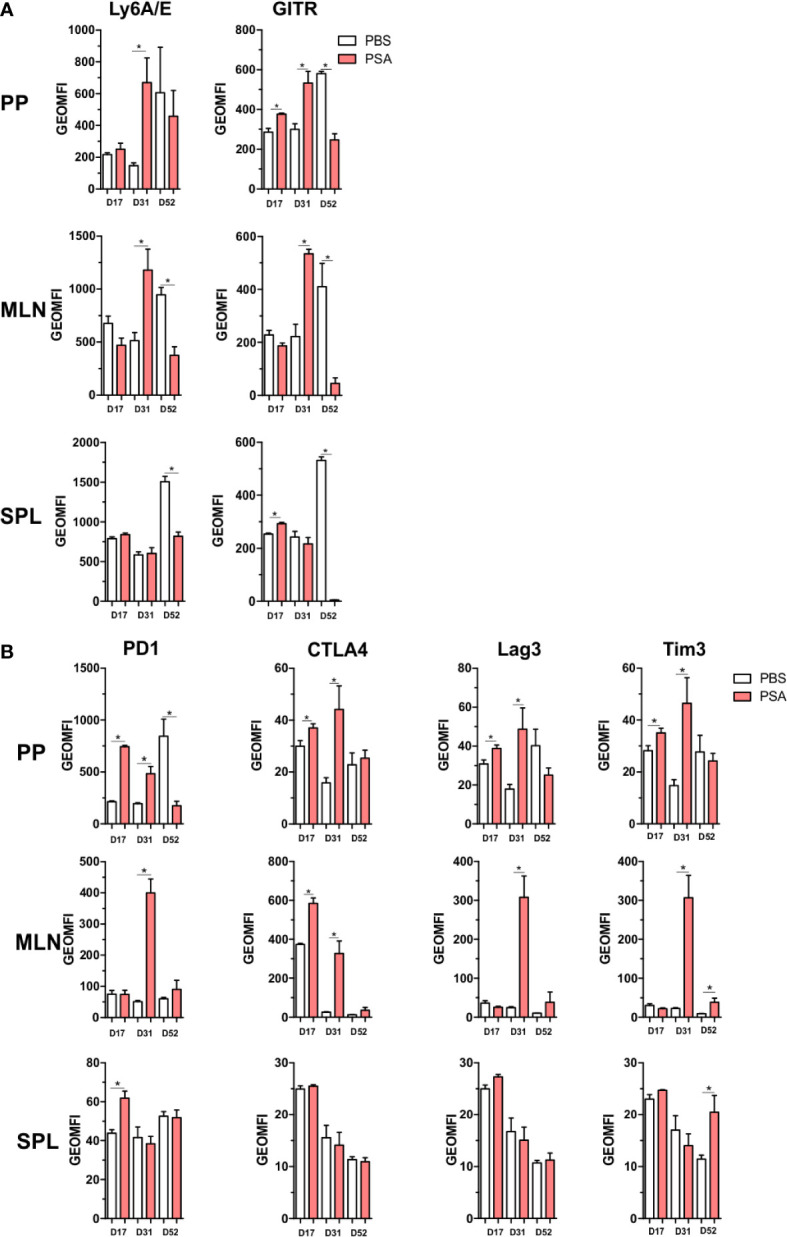
Gut associated lymphoid tissue enrichment of PSA induced markers. Mice were orally gavaged with 100 µg of PSA dissolved in PBS every 72 h, 5 gavages total. Peyer’s patches, mesenteric lymph nodes and spleens were collected on D17, D31 or D52 after initial gavage. Data is geometric MFI. **(A)** *In vivo* activation marker expression across organs and time points. **(B)** Expression profile of immunomodulatory markers across organs and time points. (n = 3 mice per group, significance of P ≤ 0.05 = *).

All of the previous analyses indicate that exposure to PSA will lead to expression and production of IFNγ and a host of IFNγ-stimulated molecules, including co-regulatory receptors associated with immune suppression but not previously associated with PSA. In order to both validate the increased expression of these molecules, and to determine where they accumulate *in vivo*, CD4^+^ cells from mice orally exposed to PSA to mimic colonization with its source bacterium *B. fragilis* were harvested directly after the last PSA gavage (D17), 2 weeks after the last gavage (D31) or 5 weeks after the last gavage (D52). We found that our *in vitro* data was replicated *in vivo* in that the expression of activation markers Ly6A/E and GITR were increased on D31 in the gastrointestinal tract-associated lymphoid tissue (GALT), including both Peyer’s patches (PP) and mesenteric lymph nodes (MLN) (**Figure 7A**). This trend was also seen for PD-1, CTLA4, Tim3, and Lag3 expression, with increased expression in the GALT on D31, compared to any other time point (**Figure 7B**). Changes in the spleen were mild and generally failed to reach statistical significance, reflecting the potent local response to PSA when administered orally and validating the extensive *in vitro* analyses herein.

## Discussion

*B. fragilis* and PSA have been the focus of research for several decades, originally due to the fact that *B. fragilis* was the most common anaerobic clinical isolate from peritoneal abscesses downstream of sepsis (44, 45). This early work robustly demonstrated the potent pro-inflammatory properties of PSA, including T cell-dependent formation of abscesses (10, 46) and adhesions (47). However, years of subsequent research revealed that *B. fragilis* and particularly PSA could also prevent abscess and adhesion formation in a T cell-dependent fashion (18, 48). It is now clear that PSA can drive immunologic inhibition of a host of inflammatory diseases such as IBD (20), asthma (11), EAE (7), and others, thereby serving as a molecular exemplar for the hygiene hypothesis (49, 50) in which the microbiota educates and biases the immune system toward health and homeostasis. These data highlight the duality of the response that was characterized at the molecular level to show IFNγ production by PSA-activated T cells (14, 51), which clonally expand (21) upon recognition of MHCII-presented PSA fragments (15). Conversely, inhibition of inflammatory disease by PSA seems to require IL-10 production (12, 22), but PSA-responding T cells lacking IL-10 still inhibit disease (11). This leads to questions about how these T cells can simultaneously produce canonically pro-inflammatory cytokines like IFNγ and inhibit inflammatory disease.

In this study, we used unbiased and complementary approaches to comprehensively characterize the T cell response to PSA in mice. Based on GO analyses and in agreement with historical data on T cell proliferation in PSA responses (21), we found that PSA-responding T cells were highly metabolically active and proliferative through the upregulation of proteins involved in cell division and other key metabolic pathways. Moreover, these T cells were dominated by an interferon-mediated expression profile, including over 100 genes known to response to interferons, which matched the increased expression of Th1-associated transcription factors such as T-bet, STAT1, and STAT4. Increased secretion of TNFα, IL-6, and CXCL-10 are also consistent with a pro-inflammatory interferon-driven response.

In light of these findings and the numerous observations on the suppressive capacity of these cells, we sought potential mechanisms of immune inhibition. GSEA analyses revealed that PSA-responding T cells overall trend toward similarity with iTregs rather than conventional T cells. The genes that promote such a correlation included the regulatory T cell-associated transcription factor FoxP3. Curiously, our previously published data demonstrated that FoxP3 is not necessary for PSA-T cells to suppress inflammation (11), and that PSA T-cells actually communicate with FoxP3 Tregs to promote suppression (12, 22). This communication was shown to be dependent upon the release of IL-2 and IL-4 (22). Here, we found that both IL-2 and IL-4 are upregulated transcriptionally, but are actually reduced in culture as measured by protein concentration. We believe that these cytokines are being removed from the media by Tregs present within the bulk CD4^+^ T cell population used for these studies. This would explain the divergence between mRNA and protein levels of IL-2 and IL-4, and is consistent with our prior findings indicating FoxP3^+^ Tregs utilize these cytokines in culture (22) and release cytokines such as IL-10 when also stimulated *via* their T cell receptor.

In addition to the support of Tregs, PSA-responding T cells also express a host of co-regulatory receptors such as Tim3, Lag3 and PD-1. These molecules, also called immune checkpoints, have gained much attention due to their role in immune suppression in cancer. Here, the expression of these inhibitory molecules both *in vitro* and within the GALT of PSA-exposed mice suggest that PSA-T cells also have a direct cell-to-cell mechanism of immune inhibition. Remarkably, these molecules also fit into an interferon-skewed response since we found that all three were increased at the cell surface of IFNγ-stimulated T cells.

Overall, this study brings together over 40 years of research on the commensal bacterium *B. fragilis* and its dominant surface antigen, the carbohydrate PSA. We reveal the duality of the response through the characterization of an interferon-dominated expression profile, expression and use of Treg-supportive cytokines like IL-2 and IL-4, and the selective deployment of co-regulatory checkpoint receptors within the gut environment of PSA-exposed mice. Although it remains possible that differences in the T cell response could be seen in the context of varied APC subsets and therefore tissues *in vivo*, the PSA response nonetheless serves to illustrate the complexity and underlying elegance of the relationship between the commensal microbiota glycome and the immune system in establishing both protection from inflammation and homeostasis.

## Data Availability Statement

The raw RNAseq data has been uploaded to the GEO - GSE156042. Other raw data supporting the conclusions of this article will be made available by the authors, without undue reservation, to any qualified researcher.

## Ethics Statement

The animal study was reviewed and approved by Case Western Reserve University School of Medicine Committee on the Case and Use of Animals.

## Author Contributions

Data collection by BC, CA, and MJ. Data analysis by CA, JH, and BC. Experimental design by BC, JH, and MJ. Manuscript preparation by BC, CA, and MJ. All authors contributed to the article and approved the submitted version.

## Funding

This research was supported by the Cytometry and Imaging Microscopy Core Facility of the Case Comprehensive Cancer center (P30CA043703). This work was funded by NIH grants GM115234 to BC, AI114109 and AI007024 to MJ, AI089474 to CA, and a grant from Boehringer Ingelheim Pharmaceuticals to BC.

## Conflict of Interest

The authors declare that the research was conducted in the absence of any commercial or financial relationships that could be construed as a potential conflict of interest.

## Supplementary Material

Supplementary Figure 1 Gene enrichment and ontology analysis in response to PSA exposure. Gene ontology was conducted on differentially expressed genes (FDR > 0.05 and log2CPM > 0). **(A)** Heat map of top 500 differentially expressed (DE) genes on D7 compared to D0. **(B)** Multidimensional scaling plot of all DE genes, showing scatter of D7 and D0 samples (each dot is a replicate sample). Top 20 significantly increased (red) or decreased (blue) gene ontology terms by **(C)** Biological process **(D)** Molecular Function and E) Cellular component (values shown are −log_10_ of p value).

Supplementary Figure 2 FoxP3 expression in PSA-stimulated populations of CD4^+^ T cells. CD4^+^ T cells and MHCII^+^ APCs were co-cultured with PSA as before for 3 or 7 days with and without varied amounts of anti-IL-4 neutralizing antibody or 5 μg of an isotype control. The **(A)** FoxP3 geometric mean fluorescence intensity (i.e., amount of FoxP3 protein) and the **(B)** percent of T cells expressing FoxP3 did not change significantly.

Supplementary Figure 3 Unchanged cytokines and chemokines in PSA-exposed cell culture. Luminex and RNAseq values for cytokines and chemokines that are not impacted by PSA stimulation.

Supplementary Table 1 List of top 500 DEGs with p values. Complete list of the top 500 differentially expressed genes in CD4^+^ cells stimulated for 7 days with PSA.

Supplementary Table 2 List of IRGs with p values. List of all interferon-responsive genes and their associated p values from CD4^+^ cells stimulated for 7 days with PSA.

Supplementary Table 3 List of all LegendScreen markers and their GeoMFI values. Complete list of all LegendScreen antibody markers and their associated geometric MFI values.

## Abbreviations

DEG, differentially expressed genes; FDR, false discovery rate; PSA, polysaccharide A; IBD, inflammatory bowel disease; EAE, experimental autoimmune encephalomyelitis; IRG, interferon responsive genes; TLR2, toll-like receptor 2; Rb^Lo^Tem, CD4^+^CD45Rb^low^CD62L^−^CD44^+^FoxP3^−^ T cells; Th1, T helper type I; RNAseq, RNA deep sequencing; CPM, counts per million; MDS, multi-dimensional scaling; GO, gene ontology; GSEA, gene set enrichment analysis; TNFR, tumor necrosis factor receptor; Treg, regulatory T cells; Tc, conventional T cells; GeoMFI, geometric mean fluorescence intensity; GALT, gastrointestinal tract-associated lymphoid tissue; PP, Peyer’s patch; MLN, mesenteric lymph node; SPL, spleen; IFNγ, interferon gamma; IL, interleukin; STAT, signal transducer and activator of transcription; mRNA, messenger RNA; MHC II, class II major histocompatibility complex.
