# Supplementary figures and images for: Characterization of Polysaccharide A Response Reveals Interferon Responsive Gene Signature and Immunomodulatory Marker Expression
Source: Front Immunol. 2020 Oct 26;11:556813. doi: 10.3389/fimmu.2020.556813 (PMC7649347; doi:10.3389/fimmu.2020.556813)

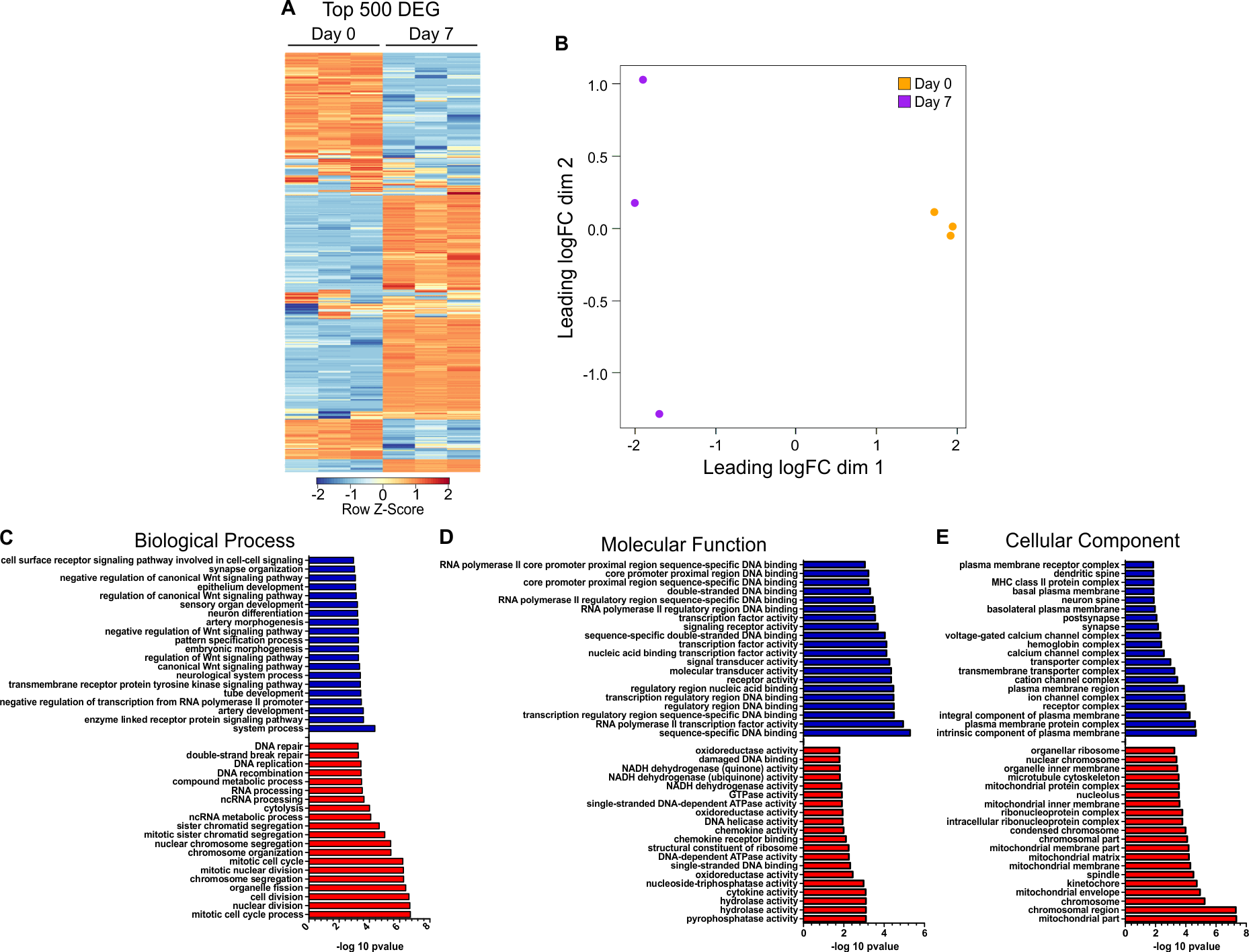

Supplement: Supplementary Figure 1 — Gene enrichment and ontology analysis in response to PSA exposure. Gene ontology was conducted on differentially expressed genes (FDR > 0.05 and log2CPM > 0). (A) Heat map of top 500 differentially expressed (DE) genes on D7 compared to D0. (B) Multidimensional scaling plot of all DE genes, showing scatter of D7 and D0 samples (each dot is a replicate sample). Top 20 significantly increased (red) or decreased (blue) gene ontology terms by (C) Biological process (D) Molecular Function and E) Cellular component (values shown are −log10 of p value). [file Image_1.tif]

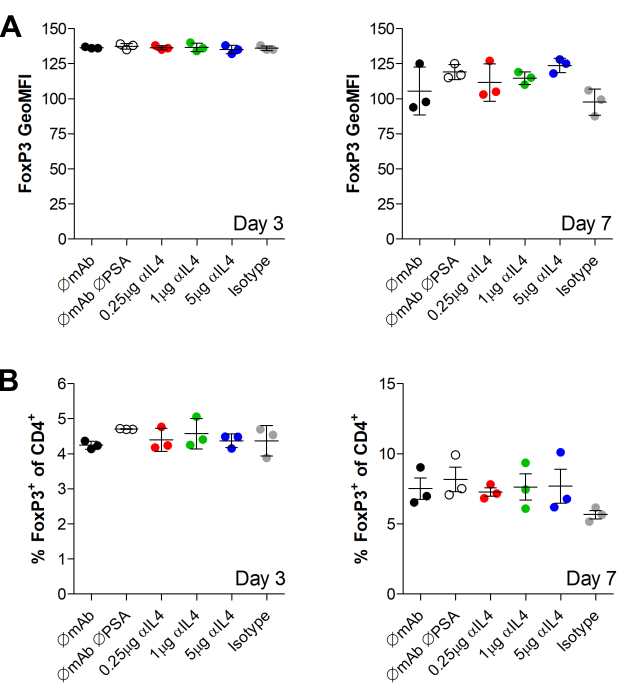

Supplement: Supplementary Figure 2 — FoxP3 expression in PSA-stimulated populations of CD4+ T cells. CD4+ T cells and MHCII+ APCs were co-cultured with PSA as before for 3 or 7 days with and without varied amounts of anti-IL-4 neutralizing antibody or 5 μg of an isotype control. The (A) FoxP3 geometric mean fluorescence intensity (i.e., amount of FoxP3 protein) and the (B) percent of T cells expressing FoxP3 did not change significantly. [file Image_2.tif]

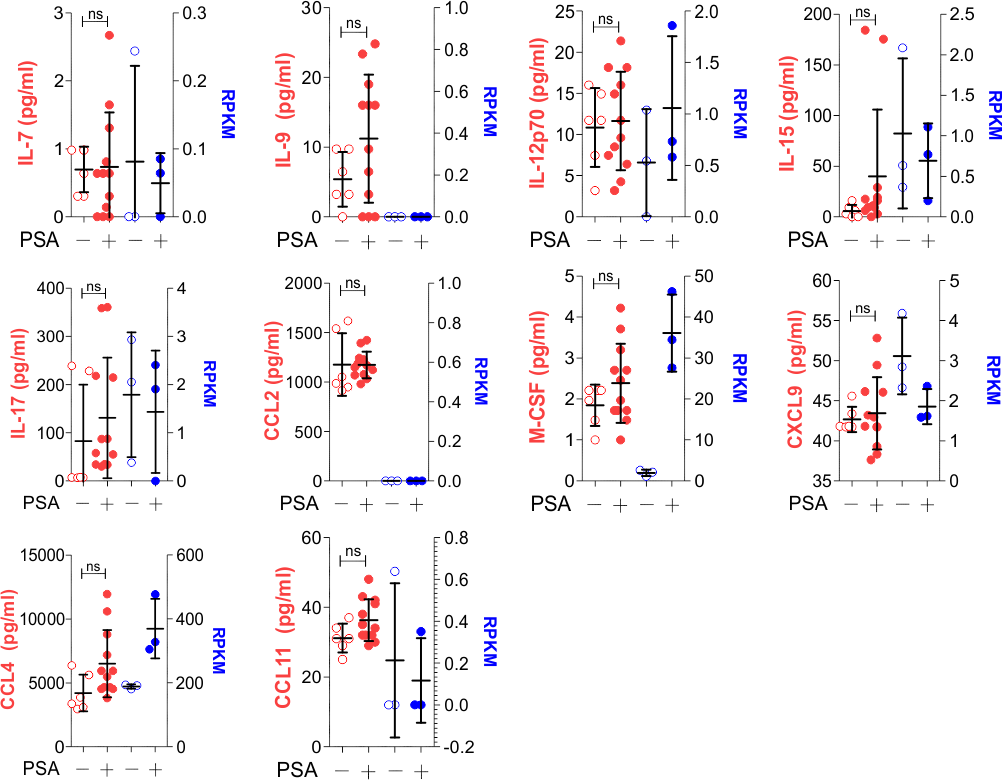

Supplement: Supplementary Figure 3 — Unchanged cytokines and chemokines in PSA-exposed cell culture. Luminex and RNAseq values for cytokines and chemokines that are not impacted by PSA stimulation. [file Image_3.tif]
